# The effect of induced fusional demand on static and dynamic stereoacuity thresholds: the digital Synoptophore

**DOI:** 10.1186/s12886-018-1000-2

**Published:** 2019-01-07

**Authors:** Laurence P. Tidbury, Anna R. O’Connor, Sophie M. Wuerger

**Affiliations:** 1 Department of Psychological Sciences, IPHS, University of Liverpool, Liverpool, UK; 2 Orthoptics, School of Health Science, University of Liverpool, L69 3GB, Liverpool, UK

**Keywords:** Fusion, Stereoacuity, Vergence, Fusion-range, Binocular vision, Synoptophore

## Abstract

**Background/aims:**

The ability to extract depth from disparity may be hindered under fusional stress, as alignment of the eyes may be more difficult to maintain consistently. Therefore we aim to determine the effect of fusional demand on stereoacuity in individuals with no known binocular vision impairments.

**Methods:**

A novel static and dynamic binocular depth detection task, capable of assessing many discrete levels of stereoacuity, was presented on digital displays attached to each tube of the Synoptophore. Stereoacuity was measured with any latent deviation fully corrected and compared to that measured at the ‘recovery’ angle. This recovery angle is where single vision is restored after decompensation to diplopia, during vergence range assessment.

**Results:**

Seventy-two subjects (50 Female, 22 Male; mean (SD) age 22 (6) years) were assessed. The amount of fusional demand was between 1 and 26 prism dioptres (PD), with a mean (SD) of 8(6)PD. Under zero fusion demand the mean (SD) static and dynamic depth detection thresholds were 322(53)” and 69(23)”. Under fusional stress these were 224(40)” and 77(21)”. There was no significant difference between thresholds in stressed and zero demand fusion (*p* = 0.08). Dynamic depth detection thresholds were significantly lower than static (*P* < 0.01).

**Conclusion:**

Fusional stress does not appear to impact on stereoacuity. The numerical value of the recovery point varied amongst individuals, but this represents a common point, where single vision is easily restored and binocularity well established. Due to individual differences in the ability to control a certain amount of fusional stress (e.g. vergences stress of 10PD, when recovery is 8PD, will perturb binocularity more than a person with a recovery of 20PD), previous reports may not accurately represent the effect of fusional stress. Whilst our findings are contrary to previous reports, we did not stress fusion beyond the recovery point and used a more accurate/repeatable method to measure stereoacuity.

## Introduction

High grade stereo acuity requires the precise alignment of the visual axis, and the sensory ability to determine the presence of binocular disparity between the left and right visual fields, and use this information to extract depth information. The binocular neurones that detect depth are sensitive to retinal information, regardless of how it is presented: [1] “if fusion is achieved, stereopsis is typically apparent” [2].

In subjects with good binocular control, the motor fusion system responds to any diplopia perceived, ensuring the visual axes are positioned on the point of fixation, resulting in zero retinal disparity at fixation. However, many individuals experience difficulty with ocular motor control, with varying impact on levels of stereoacuity, for example, the deterioration of fusional control in intermittent exotropia, can lead to in an increase in threshold [3–6]. While the consequences of a breakdown in ocular motor control are seen clinically with patients reporting a variety of symptoms resulting from the effort of maintaining binocular vision, the impact of exerting motor fusion on stereoacuity is not clear, and experiments designed to determine the importance of motor control have resulted in conflicting conclusions.

Studies have investigated the effect of fusional stress on stereopsis by simulating exodeviations of up to 40 prism dioptres (PD) and assessing stereoacuity using the Frisby Davis Distance test (FD2) and the Distance Randot® (DR) test with or without hysteresis [7, 8]. Whilst some subjects demonstrated no reduction in stereoacuity as long as no diplopia was present, findings were variable. In other subjects, fusional stress reduced the level of stereoacuity to the next banding of stereoacuity. The choice of stereoacuity test may have contributed to the variability in response, as any change may be encompassed by test/re-test variability (TRV).

The TRV value informs the clinician whether a change in stereoacuity level represents a real change in the clinical condition. This value is not known for all stereo tests but is typically over a doubling of the previous threshold measured, e.g. 50″ would need to worsen to 200″ to represent a change, with a larger increase required in poorer levels of stereoacity [9–11]. This suggests that current clinical tests may not be sensitive enough to detect a change introduced by fusional stress.

In the aforementioned studies, [7, 8] the precision of the stereoacuity measure has been lost by banding moderate stereoacuity in the range of 80–200″, and only using the 60″, 100″ and 200″ levels of the DR. Using fixed points of fusional stress also introduces variability across subjects as motor fusion varies considerably across subjects meaning that the task would be easy for some, but hard for others. The hysteresis effect allows the achievement of higher levels of motor fusion, which could be easily achieved by others without employing this.

It has been suggested that reduced stereoacuity under forced vergence may be due in part to the necessity of fine motor movements to perceive fine stereopsis being compromised by fusional demand [3]. If this were the case, it would follow that the effect would be exacerbated if the stereoscopic target was moving through depth. We have previously demonstrated that smaller amounts of binocular depth are detected in conditions where stereoscopic targets move through depth, which suggests that the changing retinal location of the stimulus does not adversely impact on acuity [12, 13].

A difficulty with previous testing has been the use of prisms to induce fusional stress. Aside from the difficulty of positioning and steadily maintaining the position of the prism, an amount of optical degradation would occur, uneven between the eyes unless the prism were split. This could contribute to any effect found. The synoptophore lends itself to maintaining a steady amount of fusional stress, with optically clear and equal optical paths, however the levels of stereoacuity testable on the device are limited in standard configuration. It is possible to modify the synoptophore, to contain computer controlled displays allowing accurate assessment of stereoacuity threshold.

This study aims to evaluate the effect of the fusional stress on stereoacuity in both static and dynamic presentations controlled with a computerised staircase procedure with the ability to present numerous levels of disparity, in subjects undergoing similar stress on fusional control.

## Materials and methods

### Subjects

Ethical approval was gained from the University of Liverpool Ethics Sub-committee and the study was performed in accordance with the ethical standards laid down in the Declaration of Helsinki. Participants were recruited from the staff and student population of the University of Liverpool. Prior to participation, informed consent was gained from each subject. Inclusion criteria for the study were for volunteers aged 16 years and over, with vision of ‘driving standard’ (0.22 LogMAR) and simultaneous perception.

### Apparatus

**Fig. 1:**
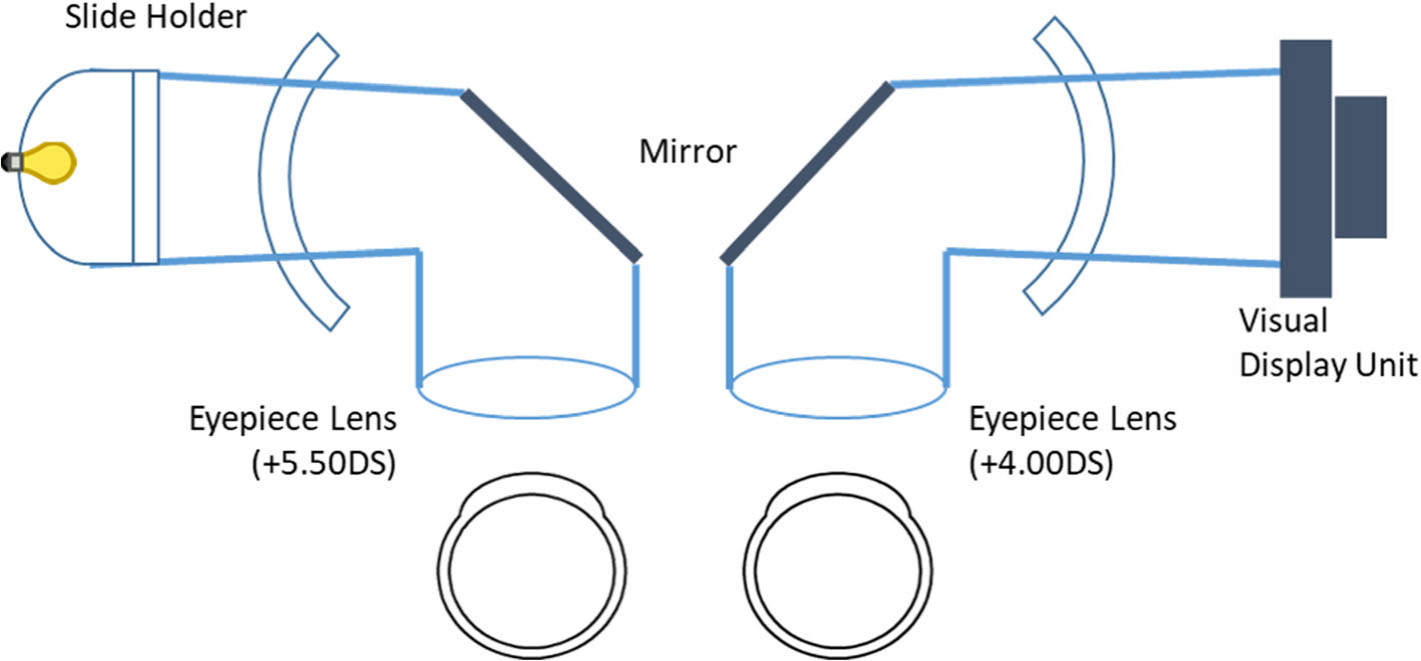
Left: the traditional layout of the synoptophore components. A translucent slide with a picture painted on one side is placed in the slide holder, retro illuminated and viewed through the mirror. Right: in place of the slide holder, a VDU has been mounted, at a slightly greater distance. Note the differing Eyepiece lens powers, which account for the difference in distance from the stimulus plane to the eye of the VDU

Stimuli were presented on a modified clinical dichoptic device: the synoptophore (Haag-Streit, Clement Clarke Ophthalmic) as shown in Fig. 1. The fixation target used in the synoptophore is typically a glass slide retro-illuminated by an incandescent bulb. In our modified device, the lamp holder/unit on each tube was replaced with two identical 1200 by 800 pixel FeelWorld 56D120175 Camera Field Monitor LCD screens run at 60 Hz. The eyepiece lens was reduced in strength from + 5.50DS to + 4.00DS, to account for the increased viewing distance. This adjustment ensured that light entering the eye was collimated, and as such maintained a zero accommodative demand. The experiment was controlled by a Pentium i3, Windows PC with an NVidia Quadro FX4600 graphics processor, running Psychopy [14]. The subject’s head rested on the forehead/chin rest integral with the synoptophore, with the eyes aligned with the centre of the screen and fixation target. The screen was positioned 0.25 m from the subject; with a horizontal resolution of 1280 pixels distributed over 0.12 m with each pixel subtending 0.021^o^ (76″). In order to increase precision, interpolation was used to create disparity steps of 0.084^o^ (19″) to be assessed, created though a shift in the luminance of the pixels at the extremes of the stimuli elements.

### Fusion

In order to determine the effect of fusional demand the positive vergence (convergence/base-out) range was used. The positive vergence range was chosen as it is least susceptible to change, especially of the recovery point [15, 16].

The objective angle of latent deviation was measured on the synoptophore using a custom fixation target in the design of a cross (with one red and one green line to each eye and a central smiley face fixation target). The objective angle was fully corrected to establish a zero fusional demand condition. After establishing the subjective breakpoint by converging the tubes using the central ABB/ADD worm screw, the tube were then diverged until a single image was reported by the subject. The recovery angle was noted and used as the fusional stress condition.

### Stimuli

*The following Stimuli, and Threshold Estimation section is similar to our previously reported method,* [12] *with selected conditions and parameters updated for use on the Synoptophore display.*

**Fig. 2:**
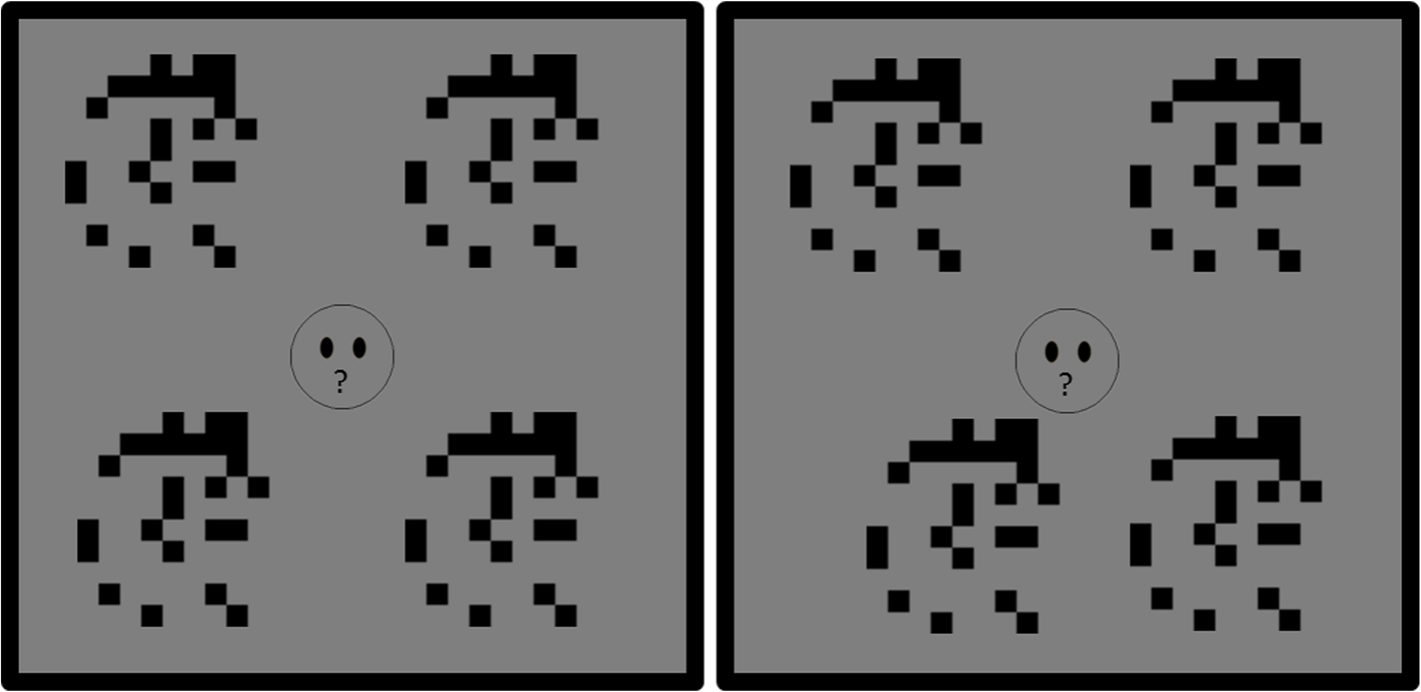
Schematic of stimuli viewed by participant. The left panel is presented to the left eye and the right to the right eye. As the lower left stimulus in the right panel has been displaced towards the fixation target, disparity has been created. If resolved this stimuli would appear forward of the fixation and control stimuli, closer to the observer. Free fusion of the above half images demonstrates the stereo effect

A four alternate forced choice (4AFC) procedure was used, with the target random dot stimulus (presented with crossed disparity compared to the screen) and three distractor stimuli (presented with zero disparity) surrounding a central fixation target with a diameter of 0.76^o^ (36 pixels) (see Fig. 2). Within each condition, the three distractor stimuli differed from the target stimulus only in the difference of lateral positions of the left and right half-images. The fixation target acted as a feedback mechanism: green colouring indicated a correct response with red indicating an incorrect response. Each stimulus subtended 2.1^o^ (100 pixel square), wherein dots of 0.21^o^ (10 pixel square) were randomly distributed with a density of 25%. The stimuli were pre-computed using Matlab (Mathworks®) and presented on a grey background with an 89% Michaelson contrast and a mean luminance of 70 cd/m^2^. The inner corners of each of the four stimuli were initially separated from the centre of the fixation target horizontally by 1.69^o^ (80 pixels) and vertically by 2.1^o^ (100 pixels). The maximum disparity level was 0.21^o^ (10 pixels) to avoid overlap of the left and right half-images of neighbouring stimuli. All stimuli were visible for a total of 1 s. This allowed the perception of smooth motion, while avoiding the perceived contrast reduction that can occur for rapidly changing patterns.

To determine the contribution of a dynamic, changing z-location stimulus to the detection of depth, two stimulus conditions were included. In each condition, the appearance of the four stimulus patches on each trial was designed to be similar, aside from the target stimulus being defined by a separation of the right and left half-images.

1. **STATIC**. Target stimuli were presented with a fixed disparity. Both the stimulus’ frontoparallel location and its dot pattern were constant throughout.
2. **DYNAMIC**. Target stimuli were presented with a disparity that changed over time (starting at zero and increasing towards the target disparity), with a constant location and dot pattern displayed. As these stimuli are seen to move laterally by some stereodeficient observers (due to substantial suppression of one eye’s input), to ensure that this percept could not be used to provide the correct answer in our 4AFC task, randomised rightward or leftward motion was added to the three distractor stimuli. The two half-images of each individual distractor stimulus moved simultaneously in the same direction and by the same distance as the target stimulus’ half images.

### Procedure

To ensure that each subject met the inclusion criteria the ETDRS logMAR chart (Precision Vision™) was used to determine if visual acuity level was better than 0.22logMAR (approximate driving standard VA) in at least one eye. Once the equipment was adjusted for inter-pupillary distance, the subject’s simultaneous perception (using the custom target), positive vergence range and any phoria was assessed on the synoptophore. If the subject had no demonstrable simultaneous perception they were excluded from testing. A trial version of the experiment was run in order to familiarise the subject with the task, this involved completing a single staircase of the STATIC and DYNAMIC in habitual primary position.

The experiment was under standard clinical lighting conditions. The subjects received standardised instructions to maintain fixation on the central target, and to use a response box (formatted in the same layout as targets on the screen) to “choose the patch that appears closest to you in space”.

### Threshold estimation

A total of four variations were tested in a blocked format; STATIC or DYNAMIC in either with or without fusional stress. Three thresholds were estimated for each condition by separate staircases (Multistair handler functional of Psychopy) [14], starting at 0.08^o^, 0.06^o^ and 0.04^o^ respectively. The initial step size was 0.04^o^, which after one reversal reduced to 0.02^o^. After a further two reversals the step size was reduced to the minimum step size of 0.005^o^. A three-down-one-up method was used so that the staircase converged to a performance of 79.4% correct [17].

To obtain depth detection thresholds for each participant, a cumulative Weibull function (Eq. 1) was fitted to the proportion of correct responses as a function of disparity level. Chance level (B) in a 4-AFC experiment is 25%, and the asymptote (A) value was set to 1. The parameters estimated were the steepness of the curve (d) and the location of the curve (c). We use c as our threshold, as this represents the disparity level at which observers achieved 72.41% correct response.

1 $$ f(x)=A-\left(A-B\right)\times \mathit{\exp}\left(-{\left(\frac{x}{c}\right)}^d\right) $$

### Statistical analysis

As a further criterion for exclusion, we used the goodness of fit value of the cumulative Weibull function; if r^2^ < 0.3 in all conditions, the subject was excluded from any analysis (for an explanation of this criteria please see reference [12]. A 2 × 2 ANOVA was performed to examine the factorial combination of the two independent variables of depth (fixed/changing) and fusional stress (zero/recovery point). Only subjects who provided a reliable response in every condition were included in the ANOVA. Pearson’s product moment correlation was performed to assess if any relationship existed between the variable angle of fusional recovery and stereoacuity levels with no fusional stress induced.

## Results

In total, 72 subjects (50 Female, 22 Male; mean (SD) age 22 (6) years) were assessed all of which completed the screening tasks successfully. Of these, 11 were excluded on the basis of unreliable performance in all conditions (unable to provide even one threshold at a performance better than chance, demonstrating an inability to perform the task).

Of the remaining subjects, Visual acuity was (mean(SD)) − 0.04(0.11) RE and − 0.05(0.12) LE LogMAR. The amount of fusional demand induced (the recovery point) was between 1 and 26 prism dioptres (PD), with a mean (SD) of 8(6)PD. The number of subjects able to perform reliably in each condition varied, details and mean thresholds for the conditions are shown in Table 1.

**Table 1:** Number of subjects who provided a reliable threshold in each condition (*n* = 61) Mean(SEM) thresholds are provided in italics

	STATIC	DYNAMIC
Zero Fusional Demand	41 *322″(53″)*	37 *69″(23″)*
Fusional Stress (recovery point)	40 *224″(40″)*	45 *77″(21″)*

**Fig. 3:**
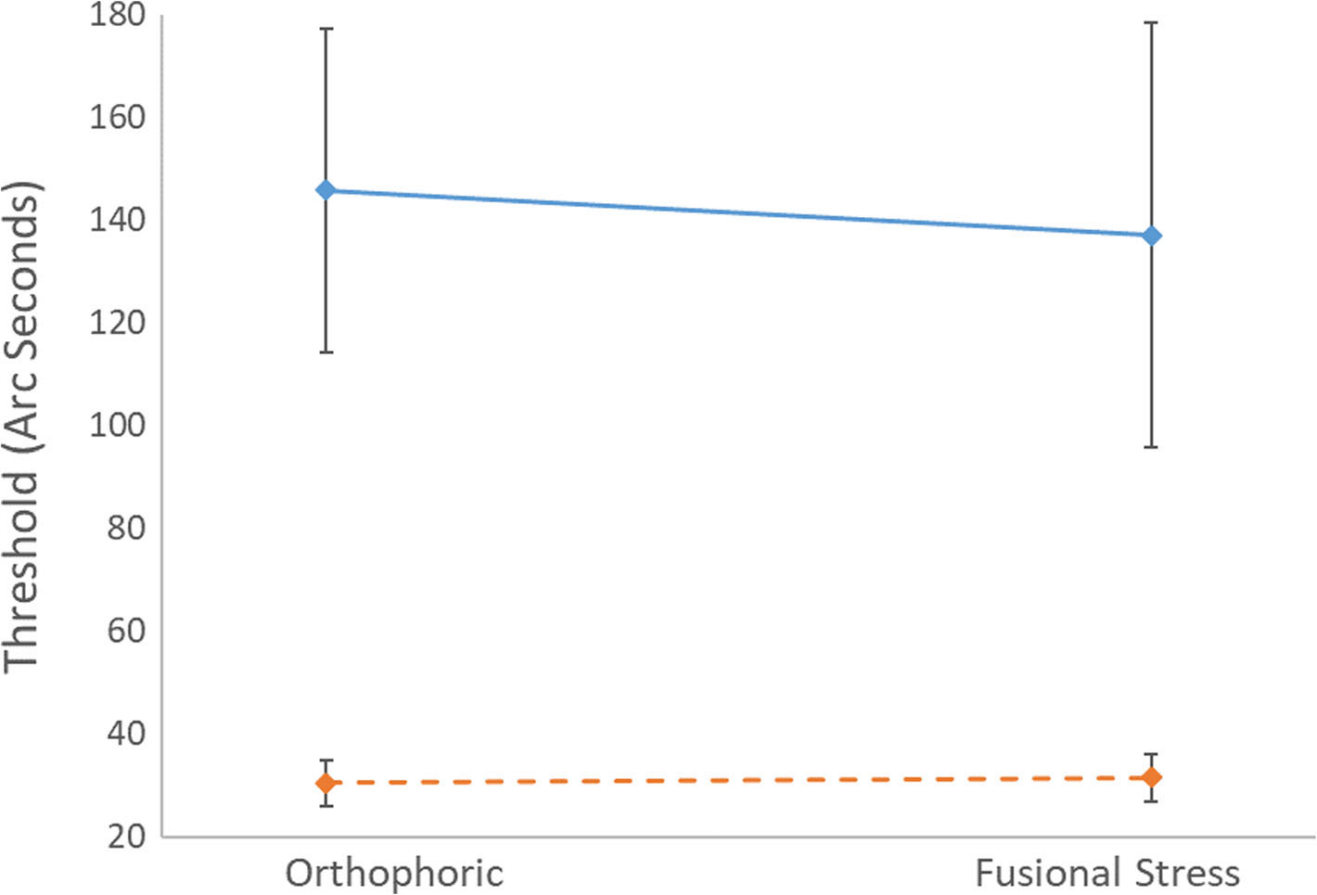
Factorial combination of disparity and pattern factors. Error bars represent ±1SEM, the solid line is the static condition and the dashed line is the dynamic condition. *N* = 21

Of the 61 subjects able to provide a reliable threshold in at least one condition, 21 were able to provide a reliable threshold in every condition tested and were included in the two way ANOVA (Fig. 3). Thresholds were lower in the DYNAMIC conditions, confirmed by the presence of a statistically significant main effect of changing depth (F(1,104) = 8.23, *p* = 0.005). No main effect of fusional demand was found (*p* = 0.40), as indicated by the similar thresholds for the two plots. The interaction between depth and fusional demand was not significant (*p* = 0.44).

**Fig. 4:**
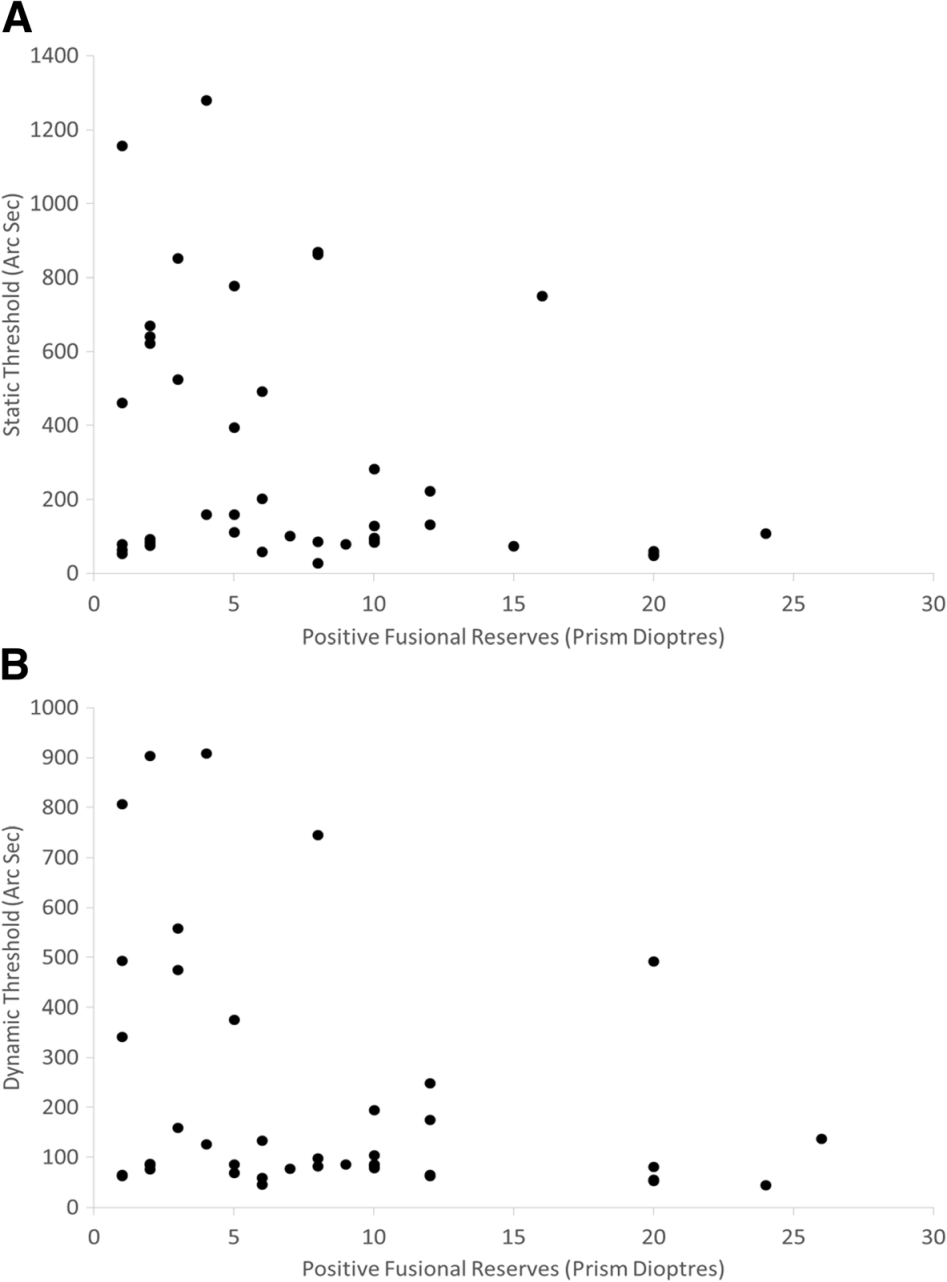
Plot of disparity detection thresholds (under zero fusional stress) versus positive fusional reserves available. A: Static condition (*n* = 41) B: Dynamic condition (*n* = 37). *N.B. there are a number of points that overlap*

To determine if the size of the fusional reserves available had any bearing on stereoacuity thresholds, a Pearson’s correlation was run between the size of the recovery angle and the static and dynamic stereoacuity thresholds with zero fusional stress (Fig. 4). No statistically significant difference from zero was found between the size of fusional recovery angle and (log) stereoacuity, in both static (*p* = 0.06) and dynamic presentations (*p* = 0.07).

## Discussion

There is no statistically significant difference between stereoacuity thresholds in the fusional stress and zero fusional demand conditions, for both static and dynamic presentations. This indicates that fusional stress up to the recovery angle does not affect stereoacuity thresholds. Whilst the size of recovery angle varied in the population tested, the size of the recovery angle had no relationship with stereoacuity threshold measured; showing that as long as a phoria is well controlled, stereoacuity levels will not be affected by the fusional load. This supports the assertion by Worth (1901); if fusion is achieved, stereopsis is typically apparent, [2] and findings that stereoacuity is either normal or absent in intermittent exotropia. [18] We demonstrate that the presentations with changing disparity result in lower thresholds than static presentations, in line with previous findings [12, 19, 20].

Our results differ at first glance, to those found by Laird et al. where a ‘reduced’ level of stereoacuity was found in up to 92 and 56% of subjects tested with the FD2 and DR at the point where single vision occurred (the fusional recovery point) [8]. The recovery point was much higher (median (IQR) 20(4) PD) than in the present study (mean (SD) 8(6) PD), which may be due to the differing methods used. Laird et al. used prisms in free space, where peripheral cues were available to aid fusion, whereas the current study used a dissociative central target (custom Bagollini striations on the synoptophore). When the study by Laird et al. was repeated including the hysteresis effect (increasing the strength of prisms from nil up to 40 PD), fusion was maintained with good stereoacuity by all but one of the 20 subjects, until the first significant drop in stereoacuity to 200″ by 10% of subjects at 30 PD on the FD2 and 20% at 35 PD on the DR. A worsening of stereoacuity (from 60″ to 100″) was reported earlier at 6 PD (FD2) and 16 PD (DR) for one of the subjects, but as previously noted, this worsening does not represent a real change in stereoacuity using these clinical tests. [9–11] The variation between subjects who demonstrated a reduction in stereoacuity and those who did not in these studies could be attributed to variation in the impact of fusional stress. Fusional stress of 10 PD may appear to be controlled by two individuals, but the demand on vergence systems may differ. The hysteresis effect may have been employed to achieve fusion at 10 PD by one individual with a recovery or re-fusion point of 8 PD, whereas the recovery point for another individual could be 20 PD. Both of these individuals were able to fuse 10 PD, but may provide differing stereoacuity results, based on fusional demands; the individual with a re-fusion point of 8PD would have less control at 10PD, than the individual that had good control up to 20 PD. The use of the recovery angle to introduce fusional stress in the present study, aimed to control these discrepancies, using a stable point relative between individuals.

The amount of fusional demand induced based on recovery points of the fusion range were between one and 26 PD. By comparing the size of recovery angle to the baseline stereoacuity, we determined that there was no relationship between the two. This further demonstrates that stereoacuity is unaffected by well controlled fusional demand.

As the amount of disparity increases during presentation in the dynamic condition, the effect of changing the stimuli position on the retina did not result in raised thresholds, in either the stressed or unstressed condition. This suggests that it is unlikely that the visual system requires the ability to finely control ocular movements to maintain fine stereopsis as previously suggested, [3] or if it does, that fine motor control is possible if fusional stress is well controlled.

Another major factor that differed between this study and previous studies is the use of a computer controlled staircase procedure to determine stereoacuity level. In combination with the ability to present a higher number of disparity levels than available in book based clinical testing, the randomisation of the order of testing to avoid adaption bias (to the induced angle), any clinician bias and order of testing biases, the threshold measured is more reliable than that achieved with clinical testing. This allowed us to statistically compare the levels of stereoacuity directly, without banding, increasing sensitivity to any change in threshold.

These findings show that inducing stress to fusional control at the recovery limit of fusion does not result in reduced stereoacuity thresholds. Inducing fusional stress beyond this amount may result in a reduction of stereoacuity thresholds, however this would employ the hysteresis effect, which is unlikely to be accessible by individuals with intermittent distance exotropia, more likely in the case of decompensating phorias, where the limits of control are noticeably reached.

## Conclusion

This study shows that stereoacuity is not significantly different between the recovery point of the prism fusion range and orthophoria. The recovery point of the prism fusion range, represents well controlled binocular vision that allows unperturbed levels of stereoacuity.

## Abbreviations

”: Seconds of Arc
ABB: Abduction
ADD: Adduction
AFC: Alternate forced choice
ANOVA: Analysis of variance
DR: Distance Randot
ETDRS: Early treatment (of) diabetic retinopathy study
FD2: Frisby Davis Distance Test
LCD: Liquid Crystal Display
LE: Left eye
LogMAR: Logarithm of the minimal angle of resolution
PD: Prism Dioptres
RE: Right eye
SD: Standard Deviation
SEM: Standard error of the mean
TRV: test/re-test variability
VDU: Visual Display Unit
